# Endoplasmic Reticulum Stress Contributes to Indomethacin-Induced Glioma Apoptosis

**DOI:** 10.3390/ijms21020557

**Published:** 2020-01-15

**Authors:** Cheng-Yi Chang, Jian-Ri Li, Chih-Cheng Wu, Jiaan-Der Wang, Su-Lan Liao, Wen-Ying Chen, Wen-Yi Wang, Chun-Jung Chen

**Affiliations:** 1 Department of Surgery, Feng Yuan Hospital, Taichung City 420, Taiwan; c.y.chang.ns@gmail.com; 2 Division of Urology, Taichung Veterans General Hospital, Taichung City 407, Taiwan; fisherfishli@yahoo.com.tw; 3 Department of Anesthesiology, Taichung Veterans General Hospital, Taichung City 407, Taiwan; chihcheng.wu@gmail.com; 4 Department of Financial Engineering, Providence University, Taichung City 433, Taiwan; 5 Department of Data Science and Big Data Analytics, Providence University, Taichung City 433, Taiwan; 6 Children’s Medical Center, Taichung Veterans General Hospital, Taichung City 407, Taiwan; wangjiaander@gmail.com; 7 Department of Industrial Engineering and Enterprise Information, Tunghai University, Taichung City 407, Taiwan; 8 Department of Medical Research, Taichung Veterans General Hospital, Taichung City 407, Taiwan; slliao@vghtc.gov.tw; 9 Department of Veterinary Medicine, National Chung Hsing University, Taichung City 402, Taiwan; wychen@dragon.nchu.edu.tw; 10 Department of Nursing, HungKuang University, Taichung City 433, Taiwan; walice@sunrise.hk.edu.tw; 11 Department of Medical Laboratory Science and Biotechnology, China Medical University, Taichung City 404, Taiwan

**Keywords:** apoptosis, ER stress, glioma, NSAID

## Abstract

The dormancy of cellular apoptotic machinery has been highlighted as a crucial factor in therapeutic resistance, recurrence, and poor prognosis in patients with malignancy, such as malignant glioma. Increasing evidence indicates that nonsteroidal anti-inflammatory drugs (NSAIDs) confer chemopreventive effects, and indomethacin has been shown to have a novel chemotherapeutic application targeting glioma cells. To extend these findings, herein, we studied the underlying mechanisms of apoptosis activation caused by indomethacin in human H4 and U87 glioma cells. We found that the glioma cell-killing effects of indomethacin involved both death receptor- and mitochondria-mediated apoptotic cascades. Indomethacin-induced glioma cell apoptosis was accompanied by a series of biochemical changes, including reactive oxygen species generation, endoplasmic reticulum (ER) stress, apoptosis signal-regulating kinase-1 (Ask1) activation, p38 hyperphosphorylation, protein phosphatase 2A (PP2A) activation, Akt dephosphorylation, Mcl-1 and FLICE-inhibiting protein (FLIP) downregulation, Bax mitochondrial distribution, and caspases 3/caspase 8/caspase 9 activation. Data on pharmacological inhibition related to oxidative stress, ER stress, free Ca^2+^, and p38 revealed that the axis of oxidative stress/ER stress/Ask1/p38/PP2A/Akt comprised an apoptotic cascade leading to Mcl-1/FLIP downregulation and glioma apoptosis. Since indomethacin is an emerging choice in chemotherapy and its antineoplastic effects have been demonstrated in glioma tumor-bearing models, the findings further strengthen the argument for turning on the aforementioned axis in order to activate the apoptotic machinery of glioma cells.

## 1. Introduction

Glioma, particularly glioblastoma multiforme, is the worst and most aggressive central nervous system malignancy [1]. Despite advances in surgical techniques, radiotherapy, chemotherapy, targeted therapy, and immunotherapy, malignant glioma still has a poor prognosis and high recurrence rate, with a median survival time of 12–15 months and a five-year survival rate of 5–13% [2]. Clinical examination has revealed an elevated expression of Bcl-2 anti-apoptosis family proteins in malignant glioma [3,4,5], implying a crucial role of apoptosis inactivation in glioma drug resistance, poor prognosis, and recurrence. In order to meet the clinical demands of glioma patients and improve prognosis, a deeper understanding of the molecular mechanisms underlying apoptosis inactivation and/or research into strategies of apoptosis activation is imperative.

Endoplasmic reticulum (ER) stress has been implicated in the pathogenesis and apoptosis decision of malignancy [6,7]. The ER is a crucial organelle for multiple cellular processes. ER stress is induced by alterations in ER homeostasis, and overwhelming exogenous or endogenous stress can lead to inappropriate function. The physiological role of ER stress is to restore the organelle’s homeostasis; however, sustained or chronic ER stress can trigger cell death program involving apoptosis [6,7]. Malignant glioma maintains ER homeostasis with an aim to suppress ER stress-induced apoptotic cell death [8]. Accordingly, ER stress not only induces glioma cell apoptosis but also sensitizes glioma cells to apoptotic treatment [9,10,11,12]. These phenomena indicate that ER stress may be a valuable target for intervention in glioma apoptosis activation.

Although the molecular mechanisms underlying glioma malignancy remain largely unclear, it is known that genetic amplification, mutation, and translocation frequently occur and contribute to malignant processes. Among the candidate genes, cyclooxygenase 2 (COX-2) overexpression is positively associated with the pathological grade and negatively correlated with the glioma survival rate [13]. Despite conflicting results in clinical practice, COX inhibition induced by nonsteroidal anti-inflammatory drugs (NSAIDs) is still a therapeutic option for glioma treatment, either as a monotherapy or combinatory therapy [14,15,16]. Accumulating evidence indicates that COX-dependent and COX-independent mechanisms underlie the anti-neoplastic action of NSAIDs and that ER stress is an off-target of the latter [17,18].

Nonselective COX inhibitor indomethacin has a profound pro-apoptotic effect on malignancy through ER stress, mitogen-activated protein kinase (MAPK), Akt, β-catenin, C/EBP Homologous Protein (CHOP), AMP-activated protein kinase (AMPK), or Aurora B kinase [19,20,21,22,23]. Findings of in vitro and in vivo studies further indicate the anti-neoplastic effects of indomethacin against glioma, involving growth inhibition, differentiation, and apoptosis [21,24,25,26,27,28,29]. Our previous study reported that Akt inactivation through the action of ceramide and the consequence of Mcl-1/FLIP downregulation substantially contributed to indomethacin-induced glioma apoptosis [30]. To extend the scope of our previous findings, this study was conducted to determine whether ER stress plays a role in indomethacin-induced glioma apoptosis and to identify the molecular basis underlying the ER stress-activated apoptotic program.

## 2. Results

### 2.1. Indomethacin Induced ER Stress in H4 Cells

**Figure 1:**
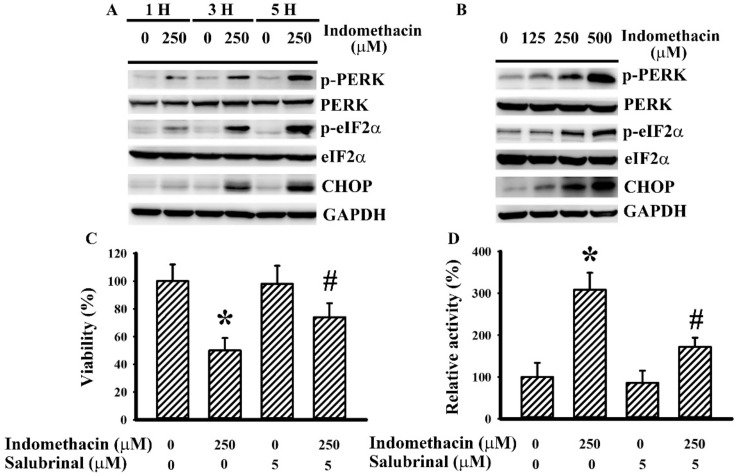
Indomethacin induced endoplasmic reticulum (ER) stress in H4 cells. H4 cells were treated with indomethacin (0 and 250 µM) over time (**A**). H4 cells were treated with various concentrations of indomethacin (0–500 µM) for 5 h (**B**). Proteins were isolated and subjected to Western blot with indicated antibodies. Representative blot of three independent experiments is shown. H4 cells were treated with indomethacin (0 and 250 µM) in the presence of salubrinal (0 and 5 µM). Cell viability (24 h) was assessed by MTS reduction assay (**C**). Caspase 3 activity (5 h) was assessed by enzymatic assay (**D**). * *p* < 0.05 vs. untreated control and # *p* < 0.05 vs. indomethacin alone control (250 µM), *n* = 3.

Typically, the phosphorylation of PRKR-like endoplasmic reticulum kinase (PERK) and eIF2α and elevation of CHOP expression are signs of ER stress [6,7]. To determine the potential involvement of ER stress in indomethacin-induced glioma cell death, parameters of ER stress were measured. Indomethacin caused elevated PERK phosphorylation, eIF2α phosphorylation, and CHOP expression in H4 cells in a time- (Figure 1A) and concentration-dependent (Figure 1B) manner. Treatment with indomethacin decreased H4 cell viability (Figure 1C) and increased caspase 3 activity (Figure 1D). Indomethacin-induced cell viability loss (Figure 1C) and caspase 3 activation (Figure 1D) were alleviated by an ER stress inhibitor salubrinal [31]. That is, ER stress appears to play a substantial role in indomethacin-induced glioma apoptotic cell death.

### 2.2. Indomethacin Altered Mitogen-Activated Protein Kinases (MAPKs) Phosphorylation in H4 Cells

**Figure 2:**
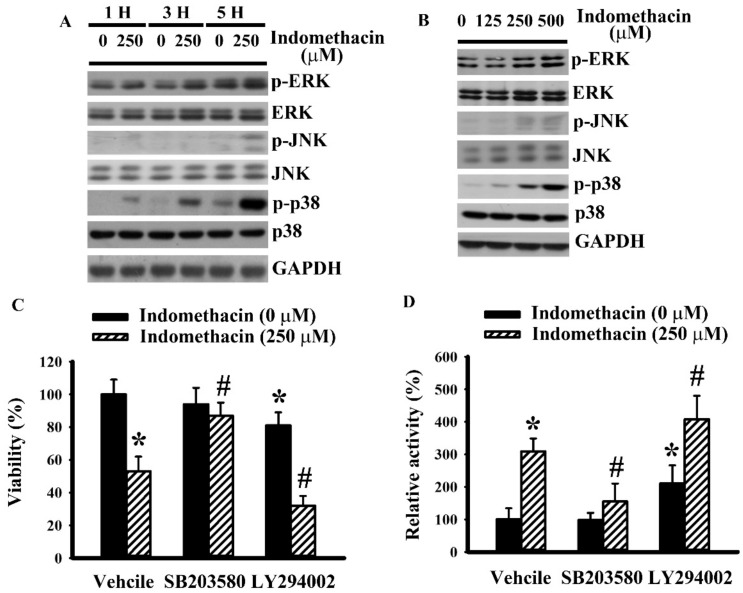
Indomethacin induced activation of intracellular signaling molecules in H4 cells. H4 cells were treated with indomethacin (0 and 250 µM) over time (**A**). H4 cells were treated with various concentrations of indomethacin (0–500 µM) for 5 h (**B**). Proteins were isolated and subjected to Western blot with indicated antibodies. Representative blot of three independent experiments is shown. H4 cells were treated with indomethacin (0 and 250 µM) in the presence of vehicle, SB203580 (0 and 20 µM), or LY294002 (0 and 20 µM). Cell viability (24 h) was assessed by MTS reduction assay (**C**). Caspase 3 activity (5 h) was assessed by enzymatic assay (**D**). * *p* < 0.05 vs. untreated control and # *p* < 0.05 vs. indomethacin alone control (250 µM), *n* = 3.

MAPKs and Akt are crucial regulators of glioma apoptosis under the control of multiple pathways, including ER stress [30,31,32,33,34]. We had already published that indomethacin caused proteolytic degradation of PARP-1 and a reduction of Akt phosphorylation in glioma cells [30]. Treatment of H4 cells with indomethacin time-dependently (Figure 2A) and concentration-dependently (Figure 2B) caused an increase of p38 phosphorylation. However, the alterations of extracellular signal-regulated kinase (ERK) and c-Jun N-terminal kinase (JNK) phosphorylation were less apparent (Figure 2A,B). To further explore the biological implications of p38 and Akt in terms of their influence on the action of indomethacin, the effects of pharmacological inhibitors of p38 and Akt were determined. p38 inhibitor SB203580 had a suppressive effect on indomethacin-induced cell viability loss (Figure 2C) and caspase 3 activation (Figure 2D). LY294002, an inhibitor of PI3K/Akt, not only caused cell viability loss (Figure 2C) and caspase 3 activation (Figure 2D) but also augmented indomethacin-induced cell viability loss (Figure 2C) and caspase 3 activation (Figure 2D). Herein, p38 hyperphosphorylation and Akt dephosphorylation were found to play an active role in indomethacin-induced glioma apoptosis.

### 2.3. p38 Mediated Indomethacin-Induced Apoptotic Execution in H4 Cells

**Figure 3:**
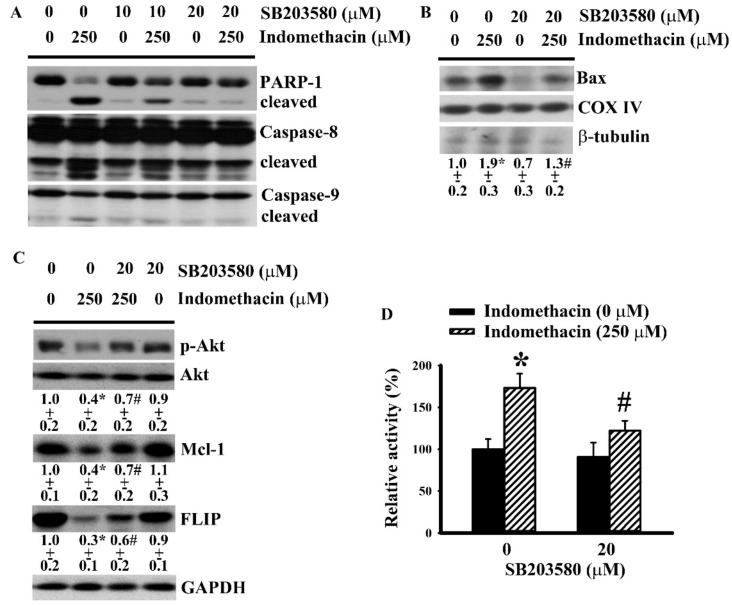
Indomethacin induced apoptotic signals in H4 cells. (**A**) H4 cells were treated with indomethacin (0 and 250 µM) in the presence of various concentrations of SB203580 (0–20 µM) for 5 h. Proteins were isolated and subjected to Western blot with indicated antibodies. H4 cells were treated with indomethacin (0 and 250 µM) in the presence of SB203580 (0 and 20 µM) for 5 h. Proteins obtained from mitochondrial fraction were subjected to Western blot with indicated antibodies (**B**). Proteins were isolated and subjected to Western blot with indicated antibodies (**C**). PP2A activity was assessed by enzymatic assay (**D**). Representative blot of three independent experiments is shown (A–C). Relative protein content was depicted under the blots (B and C). * *p* < 0.05 vs. untreated control and # *p* < 0.05 vs. indomethacin alone control (250 µM), *n* = 3.

Our previous study identified an apoptotic cascade of the axis of PP2A/Akt/Mcl-1 and FLIP in indomethacin-induced glioma apoptosis [30]. The potential link of p38 and the identified apoptotic axis in indomethacin-induced glioma apoptosis were examined. Biochemical studies revealed proteolytic degradation of caspase 8 and caspase 9 (Figure 3A) as well as mitochondrial translocation of Bax (Figure 3B) in indomethacin-treated H4 cells. In parallel, indomethacin caused a reduction of Akt phosphorylation, Mcl-1 expression, and FLIP expression (Figure 3C), but caused an increase of PP2A activity (Figure 3D). All of the indomethacin-induced biochemical alterations were alleviated by SB203580 (Figure 3A–D). The findings suggest that p38 represents an alternative machinery in mediating indomethacin-induced glioma apoptosis lying upstream of the PP2A/Akt/Mcl-1 and FLIP axis.

### 2.4. ER Stress Had a Role in Indomethacin-Induced p38 Phosphorylation in H4 Cells. 

**Figure 4:**
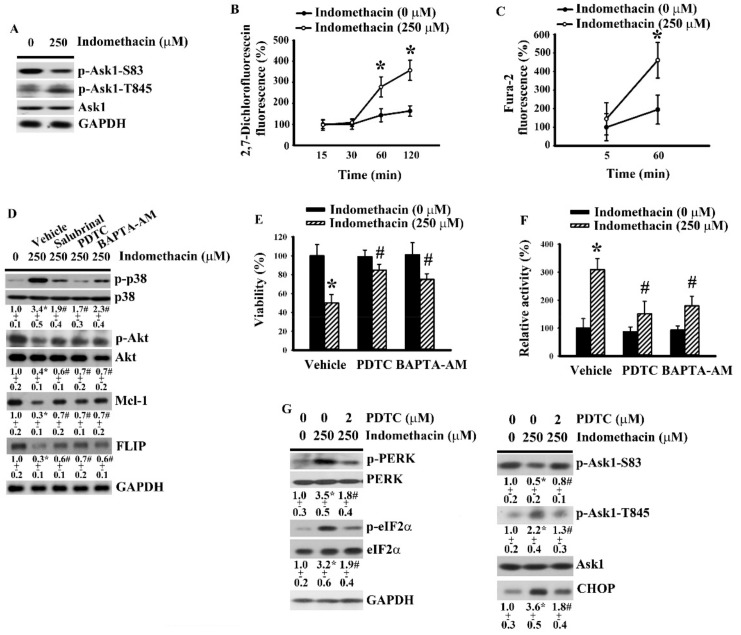
Indomethacin induced ER stress-related apoptotic signals in H4 cells. (**A**) H4 cells were treated with indomethacin (0 and 250 µM) for 5 h. Proteins were isolated and subjected to Western blot with indicated antibodies. H4 cells were treated with indomethacin (0 and 250 µM) over time. The level of reactive oxygen species was measured by 2′,7′-dichlorofluorescein fluorescence (**B**), and cytosolic Ca^2+^ concentration was determined by fura-2-acetoxymethyl ester (Fura-2AM) measurement (**C**). (**D**) H4 cells were treated with indomethacin (0 and 250 µM) in the presence of vehicle, salubrinal (5 µM), PDTC (2 µM), or BAPTA-AM (5 µM) for 5 h. Proteins were isolated and subjected to Western blot with indicated antibodies. H4 cells were treated with indomethacin (0 and 250 µM) in the presence of vehicle, PDTC (2 µM), or BAPTA-AM (5 µM). Cell viability (24 h) was assessed by MTS reduction assay (**E**). Caspase 3 activity (5 h) was assessed by enzymatic assay (**F**). (**G**) H4 cells were treated with indomethacin (0 and 250 µM) in the presence of PDTC (2 µM) for 5 h. Proteins were isolated and subjected to Western blot with indicated antibodies. Representative blot of three independent experiments is shown (A, D, and G). Relative protein content was depicted under the blots (D and G). * *p* < 0.05 vs. untreated control and # *p* < 0.05 vs. indomethacin alone control (250 µM), *n* = 3.

ER-associated Ask1 is an upstream kinase of p38 [35]. Thus, the crosstalk between ER stress and p38 axis was investigated. Indomethacin decreased serine-83 phosphorylation in inhibitory residue and increased threonine-845 phosphorylation in activating residue of Ask1 (Figure 4A). Additionally, the generation of reactive oxygen species and mobilization of cytosolic Ca^2+^ are two other ER stress-associated biochemical changes [31]. Indomethacin induced the generation of reactive oxygen species (Figure 4B) and an elevation of cytosolic free Ca^2+^ (Figure 4C) in H4 cells. As with cell viability (Figure 1C) and caspase 3 activity (Figure 1D), salubrinal reversed indomethacin-induced p38 hyperphosphorylation, Akt dephosphorylation, Mcl-1 downregulation, and FLIP downregulation (Figure 4D). Common antioxidant PDTC and calcium chelator BAPTA-AM mimicked the actions of salubrinal to reverse indomethacin-induced p38 hyperphosphorylation, Akt dephosphorylation, Mcl-1 downregulation, FLIP downregulation (Figure 4D), cell viability loss (Figure 4E), and caspase 3 activation (Figure 4F). Moreover, PDTC further reversed indomethacin-induced PERK hyperphosphorylation, eIF2α hyperphosphorylation, Ask1 serine-83 dephosphorylation, Ask1 threonine-845 hyperphosphorylation, and CHOP elevated expression (Figure 4G). The current findings indicate that the oxidative stress/ER stress/Ask1/p38 cascade contributes to indomethacin-induced glioma apoptosis, at least in part, by exerting an effect on the axis of PP2A/Akt/Mcl-1 and FLIP.

### 2.5. Indomethacin Induced Apoptotic Program in U87 Glioma Cells. 

**Figure 5:**
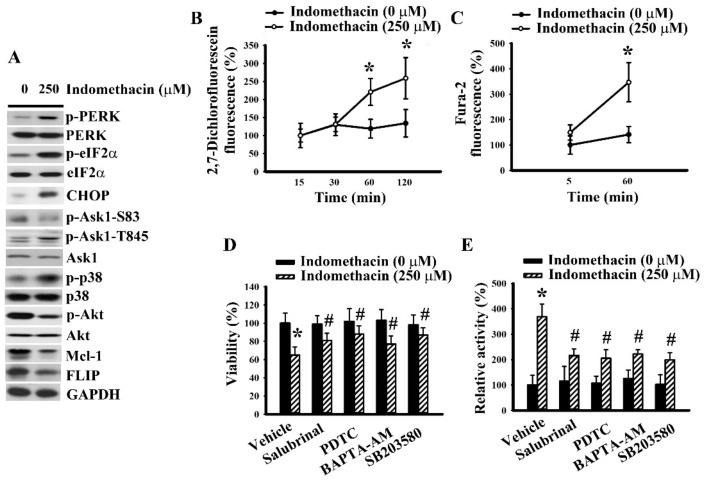
Indomethacin induced apoptosis in U87 cells. (**A**) U87 cells were treated with indomethacin (0 and 250 µM) for 5 h. Proteins were isolated and subjected to Western blot with indicated antibodies. U87 cells were treated with indomethacin (0 and 250 µM) over time. The level of reactive oxygen species was measured by 2′,7′-dichlorofluorescein fluorescence (**B**), and cytosolic Ca^2+^ concentration was determined by Fura-2AM measurement (**C**). U87 cells were treated with indomethacin (0 and 250 µM) in the presence of vehicle, salubrinal (5 µM), PDTC (2 µM), BAPTA-AM (5 µM), or SB203580 (20 µM). Cell viability (24 h) was assessed by MTS reduction assay (**D**). Caspase 3 activity (5 h) was assessed by enzymatic assay (**E**). Representative blot of three independent experiments is shown (A). * *p* < 0.05 vs. untreated control and # *p* < 0.05 vs. indomethacin alone control (250 µM), *n* = 3.

To further highlight the effect of the identified apoptotic cascade commonly seen in glioma cells, another glioma cell line, U87, was investigated for comparison. As with H4 cells, U87 cells responded to indomethacin by increasing PERK, eIF2α, Ask1 threonine-845, and p38 phosphorylation, decreasing Ask1 serine-83 and Akt phosphorylation, upregulating CHOP expression, downregulating Mcl-1 and FLIP expression (Figure 5A), and elevating reactive oxygen species generation (Figure 5B) and cytosolic free Ca^2+^ concentration (Figure 5C). Inhibitors or chelators of reactive oxygen species, ER stress, Ca^2+^, and p38 all alleviated indomethacin-induced U87 cell viability loss (Figure 5D) and caspase 3 activation (Figure 5E). That is, indomethacin was shown to have a common apoptotic cascade in both glioma cell lines.

## 3. Discussion

The dormancy or inactivation of cellular apoptotic machinery has been demonstrated to be a crucial factor in therapeutic resistance, recurrence, and poor prognosis among patients with malignant glioma [3,4,5]. Increasing evidence indicates that NSAIDs have chemopreventive effects and suggests a novel utility of indomethacin as a chemotherapeutic for killing glioma cells through distinct types of apoptotic programs [21,24,25,26,27,28,29]. Previously, we showed that the ceramide/PP2A/Akt axis, which induces Mcl-1 and FLIP downregulation, is a COX-independent target for indomethacin and turns on the apoptotic program in glioma cells [30]. Herein, we further demonstrated that indomethacin is capable of inducing oxidative stress and ER stress, as well as Ask1 and p38 activation in glioma cells. Data from mechanistic studies further indicated oxidative stress/ER stress/Ask1/p38 cascade is an alternative regulator of the PP2A/Akt axis, resulting in Mcl-1 and FLIP downregulation and eventually glioma apoptosis. Therefore, the chemopreventive effects of indomethacin against glioma are mediated, at least in part, through apoptosis activation involving ER stress.

Apoptotic cell death is an emerging factor in malignancy decision in response to therapy, and mitochondria play a central role in the coordination of pro-apoptotic and anti-apoptotic networks. The mobilization of mitochondria-related pro-apoptotic mediators is strictly controlled by pore channels formed by Bax. The formation of Bax pore channel and mitochondrial permeability are counterbalanced by the two opposite Bcl-2 family proteins, with anti-apoptotic or pro-apoptotic potential. Additionally, the cluster of membrane-associated death receptors also plays a role in initiating caspase cascades and apoptosis [36,37]. Decreased expression of Bax and BH3-only pro-apoptotic Bcl-2 family proteins or increased expression of Bcl-2 and Mcl-1-related anti-apoptotic Bcl-2 family proteins predicts poor clinical outcome in patients with malignant glioma [3,38,39,40]. We previously reported that indomethacin-induced glioma apoptosis was rarely accompanied by protein level change of Bax, Bad, Bid, and Bcl-2. Instead, the demise of Mcl-1 and FLIP expression involving Akt inactivation and Bax mitochondrial translocation and oligomerization led to substantial glioma apoptosis [30]. Findings indicate that free of Bax from Mcl-1-mediated sequestration, promotion of Bax conformational change favoring mitochondrial translocation and oligomerization, and relief of caspase 8 inhibition are targets for indomethacin-induced glioma apoptosis. Akt is crucial for transcriptional activation of Mcl-1 and FLIP and its constitutive activation predicts poor prognosis in glioma patients [41,42,43]. The activity of Akt is counter-regulated by kinases and phosphatases, particularly PI3K and PP2A. The ceramide/PP2A/Akt axis and consequences of Mcl-1 and FLIP downregulation constitute the mode of action in indomethacin-induced glioma apoptosis [30]. Herein, we further identified p38 MAPK as an alternative regulator of the PP2A/Akt axis. The inhibition of p38 by SB203580 alleviated indomethacin-induced PP2A activation, Akt dephosphorylation, Mcl-1 and FLIP downregulation, caspase 8 and caspase 9 proteolytic degradation, Bax mitochondrial translocation, caspase 3 activation, and cell viability loss. Evidence shows p38 is capable of suppressing H_2_O_2_-activated ERK signaling through activation of the PP2A [44]. Furthermore, p38 promotes Bax mitochondrial translocation through direct Bax phosphorylation [45]. Studies of relevant p38 biological activities and our current findings highlight a promising role of p38 in transducing the apoptotic signals of indomethacin in glioma cells involving the PP2A/Akt axis to turn on Mcl-1/FLIP-guided mitochondria- and death receptor-mediated apoptotic cascades and phosphorylation-triggered Bax mitochondrial translocation. The substantial involvement of the PP2A/Akt axis in the apoptotic action of indomethacin was explored and confirmed by the current findings and our previous report [30]. However, the latter hypothesis remains to be investigated because Bax phosphorylation was not measured. Although the crosstalk between ceramide and p38 has been reported [46], their potential interplay in indomethacin-treated glioma cells was not explored in this study.

p38 is a member of the MAPK family whose activities are promoted by upstream kinases and oxidative stress and inhibited through phosphatases [44,47]. The increased p38 phosphorylation in indomethacin-treated glioma cells was accompanied by concurrent elevation of PP2A activity, reactive oxygen species generation, and Ask1 activity. Intriguingly, the responses of the other two MAPK members, ERK and JNK, to indomethacin were far less than that observed with p38. Despite the discrepancy, biochemical findings suggested a potential involvement of ER stress in the regulation of p38 signaling since the MAPK upstream kinase Ask1 and free radicals correlated well with ER stress [31,32,35]. This was confirmed by our subsequent findings that showed indomethacin increased protein phosphorylation of PERK and eIF2α, and protein expression of CHOP, indicating the induction of ER stress. ER stress inhibitor alleviated indomethacin-induced p38 hyperphosphorylation, Akt, dephosphorylation, Mcl-1/FLIP downregulation, caspase 3 activation, and glioma cell viability loss.

The generation of reactive oxygen species and mobilization of cytosolic free Ca^2+^ are consequences of ER stress, and oxidative stress also acts as an inducer of ER stress [31,32,35]. Elevated levels of reactive oxygen species and cytosolic free Ca^2+^ were found in indomethacin-treated glioma cells. Their active involvement in indomethacin-altered p38, Akt, Mcl-1, FLIP, caspase 3, and glioma cell viability was demonstrated by the reversal effects of PDTC and BAPTA-AM. Furthermore, oxidative stress may also lie upstream of ER stress because of the suppressive effects of PDTC on parameters of ER stress and Ask1. Evidence shows that mitochondrial dysfunction leads to the generation of indomethacin-triggered reactive oxygen species [48]. Although our findings highlighted a crucial role of reactive oxygen species in indomethacin-induced ER stress and apoptotic execution in glioma cells, the sources of the generation of reactive oxygen species were not addressed in the current study.

The regulatory mechanisms of the stress kinase Ask1 are multifactorial. ER stress and oxidative stress promote Ask1 activity through phosphorylation on the stimulating amino acid residue (threonine-845), while Akt phosphorylates at the inhibitory moiety (serine-83) to silence its activity [49,50]. Glioma cells treated with indomethacin increased Ask1 threonine-845 phosphorylation and decreased serine-83 phosphorylation. The changes of Ask1 phosphorylation patterns were accompanied by ER stress, oxidative stress, and Akt dephosphorylation. Thus, indomethacin adopts a panel of biochemical events for the activation of Ask1 leading to glioma apoptosis by promotion of a stimulating mechanism and suppression of inhibitory machinery. However, it is important to bear in mind that Ask1 is a common upstream kinase for p38 and JNK. Besides p38 signaling, CHOP, death receptor 5, and Noxa are also downstream effectors of Ask1 and, thus, contribute to the control of cell apoptosis [22,49,50]. Currently, the refractory responses of JNK signaling and additional apoptotic mediators in indomethacin-induced glioma apoptosis have yet to be explored.

## 4. Materials and Methods 

### 4.1. Cell Cultures

Human U87 MG glioblastoma (ATCC HTB-14) and H4 neuroglioma (ATCC HTB-148) cells were cultured in Dulbecco’s modified Eagle medium (DMEM) containing 10% fetal bovine serum (FBS) [51]. To conduct experiments, cells were placed in DMEM containing 2% FBS.

### 4.2. Cell Viability Assay 

To measure viability, cells were seeded onto a 96-well plate. Cell viability was measured using an assay kit (CellTiter 96^®^ AQ_ueous_ Non-Radioactive Cell Proliferation Assay kit) in accordance with the manufacturer’s instructions (Promega, Madison, WI, USA).

### 4.3. Caspase 3 Activity Assay 

To measure caspase 3 activity, cells were seeded onto a 6-well plate. The protocols of cell lysis, extract preparation, and enzymatic reaction of caspase 3 activity were performed according to the Caspase Fluorometric Assay kit (BioVision, Mountain View, CA, USA) instructions. The levels of released fluorescent AMC moiety were measured with a fluorometer (E_x_ 380 nm and E_m_ 460 nm). The intensity of fluorescence signals was normalized by protein contents, and the relative activity was expressed.

### 4.4. Phosphatase Assay 

To measure protein phosphatase 2A (PP2A) activity, cells were seeded onto a 6-well plate. Cells were lysed and homogenized in buffers provided by the serine/threonine phosphatase assay kit (Molecular probes, Eugene, OR, USA). Five micrograms of cell homogenates were incubated with reaction buffers and substrates. The generated fluorescence products were quantified with a fluorometer (E_x_ 358 nm and E_m_ 452 nm).

### 4.5. Mitochondrial Protein Isolation 

The protocols of cell collection, disruption, and fractionation were conducted according to our previously reported methods [30]. Cell homogenates were sequentially centrifuged at 750× *g* for 10 min and at 10,000× *g* for 20 min at 4 °C. The resultant pellets were suspended in Laemmli SDS buffer.

### 4.6. Measurement of Reactive Oxygen Species 

To measure intracellular free radical generation, cells were seeded onto a 96-well plate. Cell permeable 2′,7′-dichlorofluorescein diacetate (5 µM) (Molecular Probes, Eugene, OR, USA) was added to the wells for 30 min. The generation of fluorescent 2′,7′-dichlorofluorescein was measured in a fluorometer with excitation/emission at 495/529 nm [51].

### 4.7. Measurement of Cytosolic Ca^2+^

To measure cytosolic Ca^2+^ concentration, cells were seeded onto a 96-well plate. Cell permeable fura-2-acetoxymethyl ester (Fura-2 AM) (4 µM) (Molecular Probes, Eugene, OR, USA) was added to the wells, and the fluorescent signals were measured in a fluorometer with dual excitation at 340 and 380 nm and the emission at 510 nm [52].

### 4.8. Western Blot

Cells were rinsed with cold phosphate-buffered saline and homogenized in Laemmli SDS buffer. After separation and transferring to the PVDF membranes, proteins on the membranes were identified with the following antibodies: poly(ADP-ribose) polymerase 1 (PARP-1), extracellular signal-regulated kinase (ERK), phospho-ERK, c-Jun N-terminal kinase (JNK), phospho-JNK, p38, phospho-p38, Akt, phospho-Akt, PERK, phospho-PERK, eIF2α, phospho-eIF2α, CHOP, caspase 8, caspase 9, Bax, cytochrome oxidase IV (COX IV), Mcl-1, FLICE inhibiting protein (FLIP), apoptosis signal-regulating kinase 1 (Ask1), phospho-Ask1 Ser-83, phospho-Ask1 Thr-845 (Santa Cruz Biotechnology, Santa Cruz, CA, USA), glyceraldehyde-3-phosphate dehydrogenase (GAPDH) (R&D Systems, Minneapolis, MN, USA), and β-tubulin (Sigma-Aldrich, St. Louis, MO, USA). The reaction products were determined with horseradish peroxidase-labeled IgG and visualized using enhanced chemiluminescence (ECL) Western blotting reagents. Finally, the signals were determined quantitatively with a computer image analysis system (IS1000; Alpha Innotech Corporation).

### 4.9. Statistical Analysis 

Data are expressed as means ± standard deviations. Statistical comparisons were analyzed using one-way analysis of variance followed by Tukey’s or Dunnett’s test. A *p* value less than 0.05 was considered statistically significant.

## 5. Conclusions

In conclusion, we found that indomethacin exhibited anti-neoplastic effects against glioma H4 and U87 cells involving apoptosis. The induction of death receptor- and mitochondria-mediated apoptotic cascades were accompanied by a series of biochemical changes, including reactive oxygen species generation, ER stress, Ask1 activation, p38 hyperphosphorylation, PP2A activation, Akt dephosphorylation, Mcl-1 and FLIP downregulation, Bax mitochondrial distribution, and caspase 3/caspase 8/caspase 9 activation. Investigations of pharmacological inhibition related to oxidative stress, ER stress, free Ca^2+^, and p38 revealed that the axis of oxidative stress/ER stress/Ask1/p38/PP2A/Akt constitutes an apoptotic cascade leading to Mcl-1/FLIP downregulation and glioma apoptosis. There are limitations to our experiments because of the lack of normal cell lines for comparison and in vivo evaluations. Since indomethacin is an emerging choice in chemotherapy and its antineoplastic effects have been demonstrated in tumor-bearing models [26,27,28], our findings support further exploration of a therapeutic approach in which the axis for the activation of apoptotic machinery in glioma cells is turned on.

## Abbreviations

AMPK	AMP-activated protein kinase
Ask1	Apoptosis signal-regulating kinase-1
JNK	c-Jun N-terminal kinase
COX IV	Cytochrome oxidase IV
DMEM	Dulbecco’s modified Eagle medium
ER	Endoplasmic reticulum
ECL	Enhanced chemiluminescence
ERK	Extracellular signal-regulated kinase
FBS	Fetal bovine serum
FLIP	FLICE-inhibiting protein
GAPDH	Glyceraldehyde-3-phosphate dehydrogenase
MAPK	Mitogen-activated protein kinase
NSAIDs	Nonsteroidal anti-inflammatory drugs
PARP-1	Poly(ADP-ribose) polymerase 1
PP2A	Protein phosphatase 2A
